# Population Dynamics of the Critically Endangered Golden Lancehead Pitviper, *Bothrops insularis*: Stability or Decline?

**DOI:** 10.1371/journal.pone.0095203

**Published:** 2014-04-22

**Authors:** Murilo Guimarães, Roberto Munguía-Steyer, Paul F. Doherty, Marcio Martins, Ricardo J. Sawaya

**Affiliations:** 1 Departamento de Biologia Animal, Instituto de Biologia, Universidade Estadual de Campinas, Campinas, São Paulo, Brazil; 2 FES-Iztacala, Universidad Nacional Autónoma de México, D.F., Facultad de Estudios Superiores Iztacala, Universidad Nacional Autónoma de México, Tlalnepantla, Estado de México, México; 3 Department of Fish, Wildlife, and Conservation Biology, Colorado State University, Fort Collins, Colorado, United States of America; 4 Departamento de Ecologia, Instituto de Biociências, Universidade de São Paulo, São Paulo, São Paulo, Brazil; 5 Departamento de Ciências Biológicas, Universidade Federal de São Paulo, Diadema, São Paulo, Brazil; Université de Sherbrooke, Canada

## Abstract

Little is known about vital rates of snakes generally because of the difficulty in collecting data. Here we used a robust design mark-recapture model to estimate survival, behavioral effects on capture probability, temporary emigration, abundance and test the hypothesis of population decline in the golden lancehead pitviper, *Bothrops insularis*, an endemic and critically endangered species from southeastern Brazil. We collected data at irregular intervals over ten occasions from 2002 to 2010. Survival was slightly higher in the wet season than in the dry season. Temporal emigration was high, indicating the importance of accounting for this parameter both in the sampling design and modeling. No behavioral effects were detected on capture probability. We detected an average annual population decrease (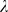 = 0.93, CI = 0.47–1.38) during the study period, but estimates included high uncertainty, and caution in interpretation is needed. We discuss the potential effects of the illegal removal of individuals and the implications of the vital rates obtained for the future persistence and conservation of this endemic, endangered species.

## Introduction

Snake populations have been declining around the world [1]–[3], and despite many years of gathering knowledge, snakes are still intriguing vertebrates difficult to study in the field. Part of such difficulty derives from their natural history characteristics, which include elusive habits, cryptic behavior, and low densities [4]–[5]. Furthermore, habitat heterogeneity and complexity of some systems such as tropical forests make species difficult to find and study. As a consequence, most information on snake population biology is still anecdotal and characterized by the use of indices and basic descriptions of vital rates [4], but see [6]. Robust quantitative population estimates including abundance and survival are generally uncommon in the literature [7].

The scarcity of reliable information in snake systems is even greater when considering field studies that account for the imperfect detectability of individuals and species [8] or that uses robust modeling techniques [5]. Fortunately, in the last decade there has been a growing number of field studies and application of novel analytical techniques leading to more accurate estimates [5], [9]–[16]. Accounting for detectability improves the estimation of population rates, including abundance and dynamics, critical for managing species [17]. Improving detection probability estimates through good sampling design and predictor variables is important because it provides support for the evaluation of all other parameters [18]. This, in turn, enables more effective management guidelines to be implemented in snake populations [5], [13].

Model-based approaches are valuable tools that can help improve quality of population studies when sampling elusive species, such as pitvipers. Pitvipers are generally cryptic snakes with life history characteristics including low reproductive frequency and site fidelity that make them vulnerable to population declines [19]. Among the new world pitvipers, the clade *Bothrops* (see [20] for a taxonomic elucidation) is distributed throughout the Neotropical region, with several threatened species [21], and usually scarce information on demography.

The golden lancehead, *Bothrops insularis* is an insular and endemic pitviper, restricted to a single island in southeastern Brazil, and is critically endangered [21]. In 2008, a concern was raised about population decline based on decreasing encounter rates and suspicion of illegal trading [22]. The same authors also reported an offer of US$ 30,000 for one specimen. Here, using mark-recapture models, we tested the hypothesis of decline of the population while accounting for detection probability, and provided vital rate estimates as the first attempt to investigate the dynamics of this threatened island pitviper.

## Materials and Methods

### Study site

*Bothrops insularis* is restricted to the Queimada Grande Island (24° 29′ 12″ S, 46° 40′ 28″ W; see detailed description in [22]), a small (43 ha) and protected reserve about 30 km from the southern coast of São Paulo state, southeastern Brazil. The island consists of bare rocky areas, open grassy areas, and lowland rainforest; the latter covers approximately 60% of the entire island [22], and is the typical habitat of the target species. Altitudinal variation in the island ranges from 0 to 200 m above sea level [22]. The climate is subtropical with two prominent seasons; one rainy and warm (October to March) and the other dry and cold (April to September, see [23] for details). Relative air humidity is above 90%. The island serves as an important migration route for passerine birds, which use the island to rest and feed and are preyed upon by the pitviper [24].

### Data collection

We used visual encounter surveys on each sampling occasion, with four to six trained observers searching for snakes on the ground and on the trees, during daylight, in a single 1370×3-meter linear transect that crosses the island in the north-south direction (see map in [22]). Due to weather and logistical restrictions, we visited the island 10 times (each field trip hereafter referred as primary sampling occasions) irregularly from 2002 to 2010 in different periods of the year (Table 1). Each visit varied from two to four days (each sampling day hereafter referred as secondary sampling occasions), resulting in 28 sampling occasions (see Table 1). Because our samples were composed almost entirely by adults (juveniles are difficult to spot in the field), we restricted our analysis only to adults, i.e., mature males (>505 mm) and mature females (>555 mm; [23]).

**Table 1:** Date, number of secondary occasions, and air temperature of each of the 10 field trips (primary occasions) between 2002 and 2010.

Primary occasion	Date	Secondary occasions	Air temperature^a^	Season^b^
1	Jan/2002	4	26.9	Summer (wet)
2	May/2002	2	22.2	Autumn (dry)
3	Dec/2004	3	27	Spring (wet)
4	Jun/2007	3	20.7	Autumn (dry)
5	Aug/2007	3	17.9	Winter (dry)
6	Mar/2008	3	26.3	Summer (wet)
7	Jul/2008	3	20	Winter (dry)
8	Oct/2009	3	21	Spring (wet)
9	Jan/2010	2	26.4	Summer (wet)
10	Mar/2010	2	26.2	Summer (wet)

a average monthly temperature (°C)

b Austral seasons

Each individual sighted was hand-captured and marked using passive integrated transponders (PIT tags) injected in the latero-posterior region of the body with sterilized needles. Pit tag/body mass ratio was never higher than 1%. We identified the sex and measured the snout-vent length (SVL) of each individual using a flexible tape (to the nearest cm). Afterwards, we released the individuals at the same place we sighted them. We also measured monthly air temperature to include in the analysis since temperature is supposed to predict the activity of ectothermic animals even in subtropical regions [25]–[26].

All procedures involving sampling and marking conducted with this endangered and protected species are in accordance with relevant national and international guidelines to ensure ethical appropriateness, for which we obtained the necessary permits from the Instituto Chico Mendes de Conservação da Biodiversidade (ICMBio, permit number 14858-2), the agency of the Brazilian Ministry of Environment responsible for the Queimada Grande Island. Additionally, no snake was killed for this study.

### Mark-recapture modeling

To estimate the population vital rates, we used the Huggins closed capture model, [27]–[28] in a Robust Design [29], to obtain maximum likelihood estimates of survival probability (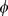), temporary emigration (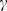), first capture (*p*) and recapture probability (*c*), using program MARK [30]. We adjusted the time interval between visits because they were irregular.

The robust design incorporates aspects of closed population and open population models. Data requirements and assumptions follow mark-recapture modeling for both closed and open populations [17]. In the former, the population is assumed to be biologically and geographically closed (neither births/deaths nor immigration/emigration are allowed) within primary sampling occasions and the model provides estimates of abundance and recapture probability. When the population is open, the model relaxes the closure assumption between primary occasions and is used to estimate survival [29]. The assumption of closure within each primary occasion in this study is based on the biology of pitvipers, which are usually sedentary and sit-and-wait predators that remain in the same foraging and resting area for many days or weeks ([31]–[33]; M.M. and M.G., pers. obs. on radio-tagged *B. atrox* and *B. insularis*). Survival was assumed to be 1.0 over the short period of the secondary sampling occasions.

Besides the estimation of 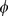, which is a product of true survival and fidelity (thus, permanent emigration and death are confounded), one advantage of the robust design is the estimation of temporary emigration, that is animal movement that leads to temporary unavailability of individuals for capture in the sampling area [34]. Since open and closed models assume that all animals are always available for capture, failing to meet this assumption may bias the estimates [34]. Temporary emigration is provided by two parameters, 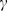
″ and 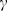
'. The parameter 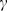
″ represents the probability that an individual is a temporary emigrant on primary occasion *t* given it was alive and available for capture on primary occasion *t* - 1. The parameter 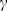
' represents the probability of an individual that was a temporary emigrant on primary occasion *t* – 1 remains as a temporary emigrant on primary occasion *t*. We used these parameters because we suspect that movement on and off the surveyed area occurred between the primary occasions.

The dissociation between the probability of the very first capture (*p*) and recapture (*c*, conditional on having been captured at least once) allows for testing positive or negative behavioral responses to the first animal encounter and the subsequent ones (i.e., trap-shy and trap-happy effects, [35]). Since we physically captured and marked individuals with PIT tags, the recapture probability could be lower due to stress, for instance. Conversely, if *p* = *c*, no behavioral response is assumed.

The Huggins closed capture robust design also provides an estimate of the population abundance (*N*) for each primary occasion as a derived parameter, which means that *N* is conditioned out of the likelihood and it is based on the estimated capture probabilities of individuals captured at least once and obtained through a Horvitz-Thompson estimator. We used the estimates of the population abundance to obtain the finite population growth rate 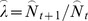 for each one of the intervals of the study, where 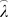 is a positive number that measures the proportional population increase/decrease from 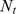 to 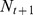. We then obtained the geometric mean growth rate for the study period, where lambda values above 1 indicate an increase in the population. We used the delta method [36] to obtain sampling variances and derive standard errors and confidence intervals for the estimated 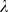.

We constrained each parameter to be a logit-linear function of individual and/or temporal covariates. We kept a simple parameterization and did not include time effects on parameters because we anticipated poor support for complex models due to our limited data set. We modeled survival as a function of sex (SEX) because we suspected there were activity differences between males and females, and season (SEASON) because of seasonal differential ectothermic activity. These behavioral differences may lead to distinct survival probabilities. We also included a model that considered survival as constant over time, denoted by a period (.). Concerning movement, we tested for a random emigration pattern among individuals (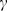
″ = 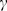
'), meaning individuals move on and off the study area randomly. Conversely, we tested for a first-order Markov process of emigration, where the state of the individual at *t* – 1 influences the state at *t*. For the detection process (capture and recapture probabilities) we tested for a positive effect of body size (snout-vent length, SVL) on capture, because larger snakes may be more detectable, monthly air minimum temperature (MINTEMP), as well as no time variation, meaning constant survival (.). Some models included the parameter *c* (recapture probability) to test for behavioral effects of capturing.

We built 64 models representing hypotheses about the effects of the covariates on the parameters running all possible additive combinations of factors that make logical sense to obtain a balanced model set [37]. Our most parameterized model (i.e., the global model) assumed that survival, initial capture probability and recapture probabilities were additive functions of a temporal covariate and an individual covariate, and allowed for Markovian time-constant temporary emigration, 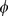
(season+sex) 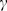
″ (.) 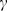
'(.) *p*(mintemp+svl) *c*(mintemp+svl). Because the robust design has no goodness-of-fit test available we tested for extra binomial variation using the median c-hat approach by collapsing secondary occasions in the context of the live encounter Cormack-Jolly-Seber model.

We ranked and selected models using Akaike’s Information Criterion [38] adjusted for small sample sizes and extra binomial variation (QAIC*c*, [39]). Estimates were model averaged in order to include model uncertainty [39] and all parameters are reported with the 95% unconditional variance confidence intervals. We calculated the relative importance of each covariate through the cumulative QAIC*c* weights [39]. Following [40], we considered covariates with cumulative QAIC*c* weight >0.5 to be important.

## Results

We marked 291 adult individuals (126 males, 165 females) and recaptured 46 of them at least once. Males averaged 588 mm in total length (range 505–718 mm) and 80 g (range 55–130 g), whereas females 715 mm (range 555–940 mm) and 180 g (range 80–480 g). We detected some extra binomial variation in the dataset (*ĉ* = 1.8) and adjusted our model set and variances accordingly. Models presented similar weights resulting in high uncertainty in model selection (Table S1).

**Figure 1:**
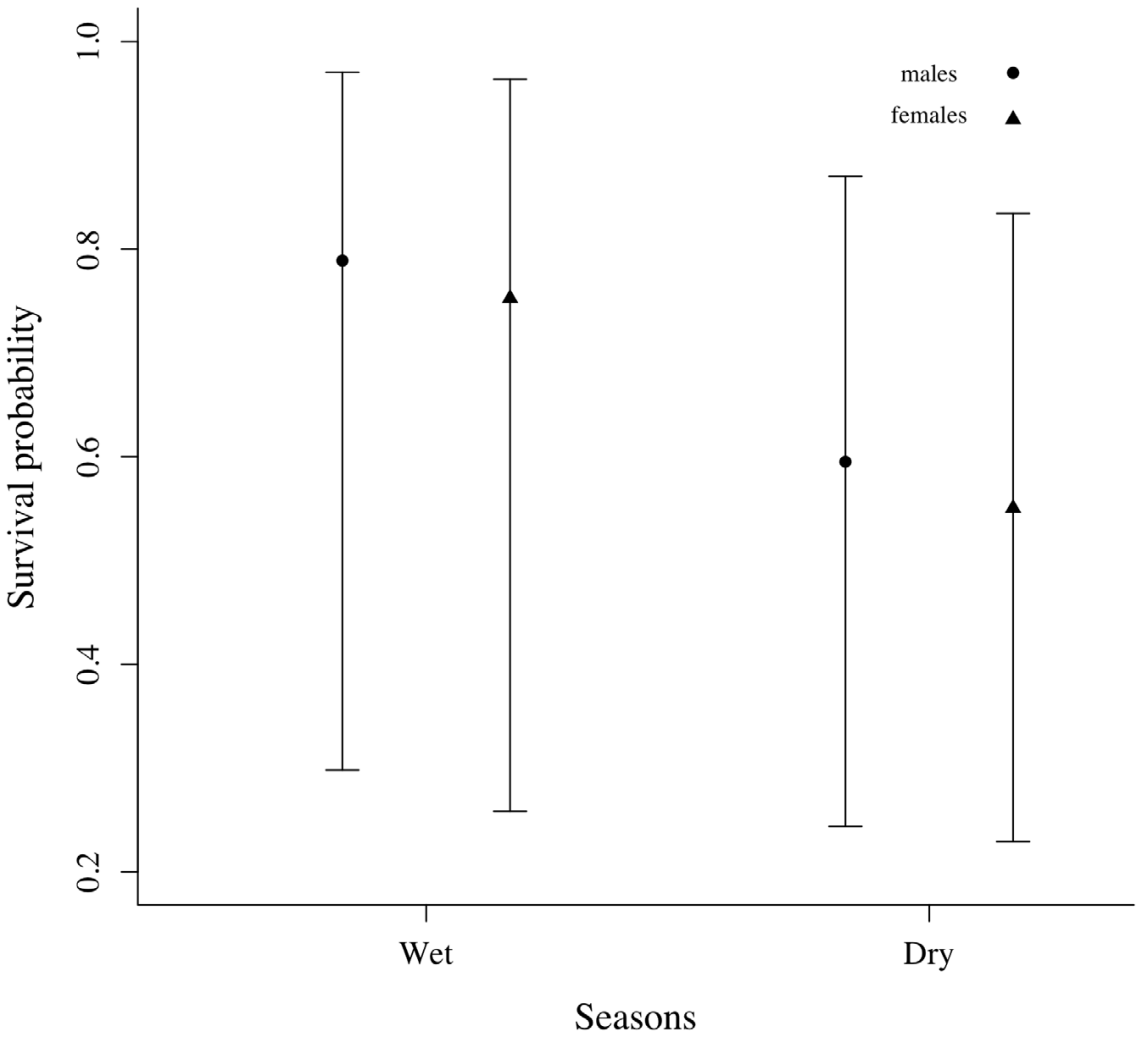
Survival probability. Seasonal survival probability and 95% confidence intervals for adult golden lancehead pitvipers, *Bothrops insularis*.

Sex was not strongly correlated with survival (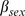 = 0.45, CI = −0.86–1.75; cumulative QAIC*c* weight of 0.33; Table 2). Season had a positive, but marginal effect on survival (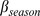 = 2.04, CI = −1.43–5.51; cumulative QAIC*c* weight of 0.51; Table 2). Slightly higher model-averaged survival estimates were found for the wet season than in the dry season for males and females (Fig. 1).

**Table 2:** Cumulative AIC*c* weights of the covariates used to model survival probability (Φ), temporary emigration (*γ*″, *γ'*) and detection probability (*p, c*).

Variable	Cumulative AIC*c* weight
Φ (season)	0.51
Φ (sex)	0.33
*γ*' (Markov process)	0.36
*p* (air temperature)	0.27
*p* (snout-vent length)	0.27
*c* (behavioral effect)	0.34

Model-averaged estimates suggested a slightly higher probability of being absent from the sampling area (an emigrant) during period *t* if the individual was an emigrant during the previous period *t* - 1, but the confidence intervals were large showing uncertainty (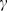
' = 0.76, CI = 0.34–0.95; 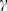
″ = 0.70, CI = 0.35–0.90). Thus, a random pattern of movement on and off the study area was more supported than a Markovian pattern (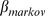 = 1.57, CI = −1.25–4.40), which presented a cumulative QAIC*c* weight of 0.36 (Table 2).

Conditional on individuals being available for detection, the first capture (0.10, CI = 0.02–0.43) and recapture probability (0.07, CI = 0.02–0.27) were similar and did not support strong behavioral effects on recapturing individuals, although we observed a small tendency of decrease (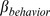 = −0.78, CI = −2.09–0.53). Minimum air temperature and body size were not important predictors in the detection process (Table 2, Table S1).

**Figure 2:**
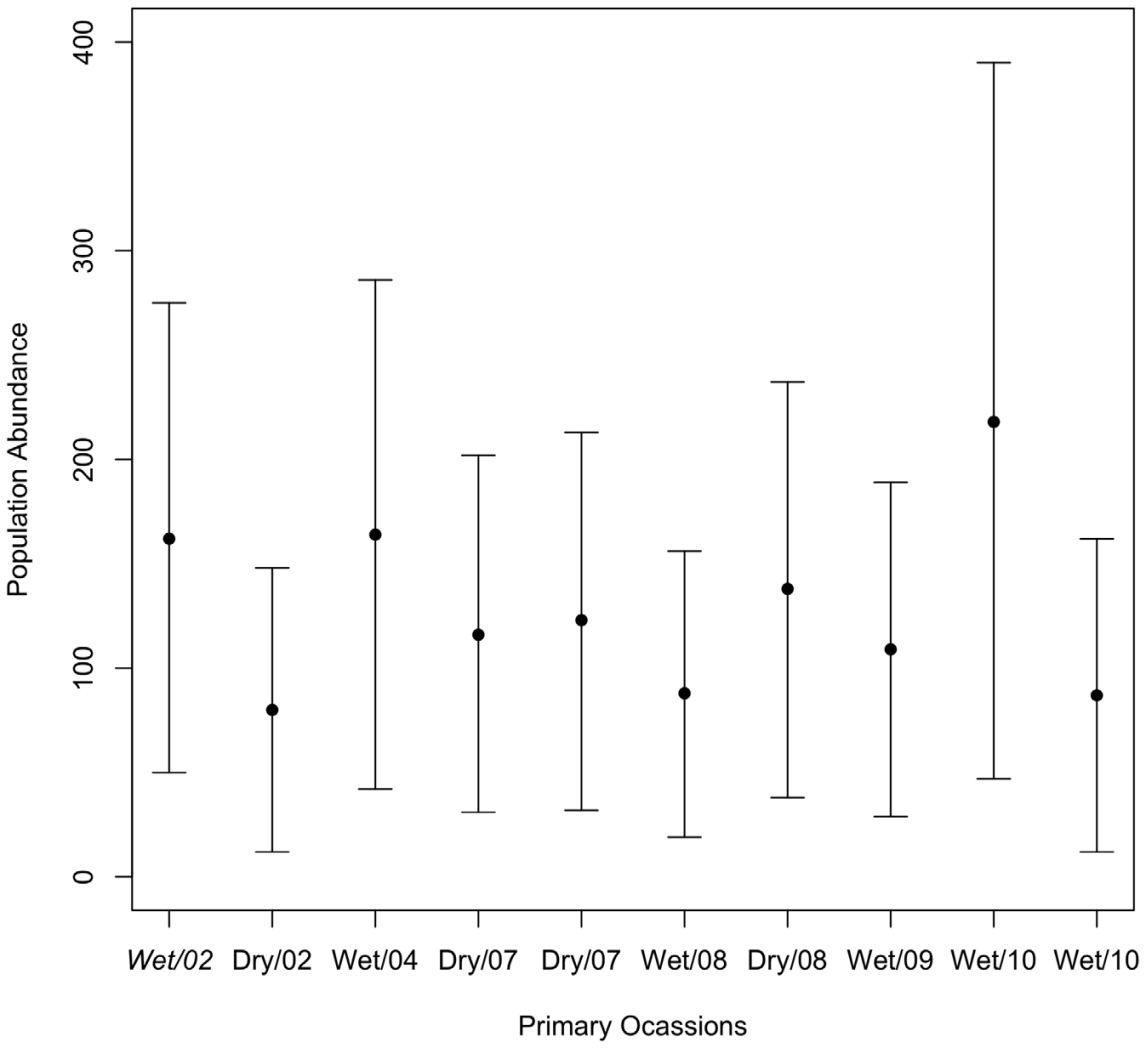
Population abundance. Population abundance and 95% confidence intervals for the golden lancehead pitviper, *Bothrops insularis* during the study.

**Figure 3:**
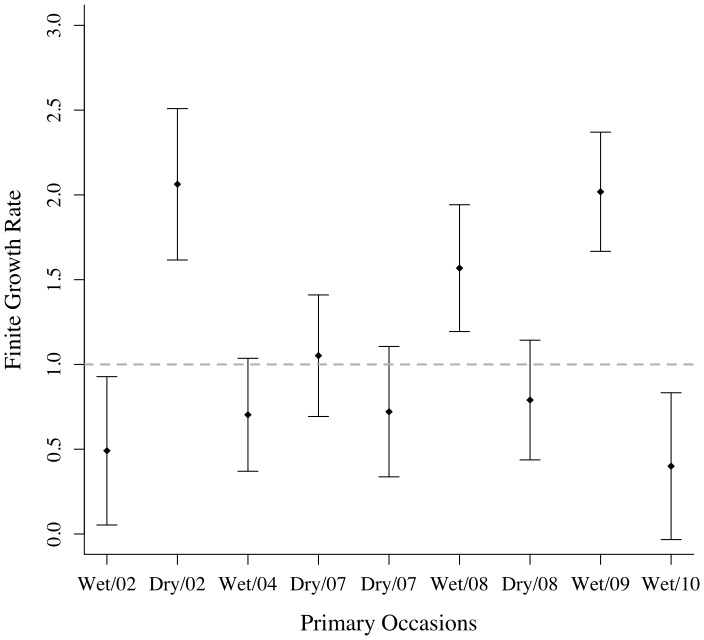
Population growth. Finite population growth rate and 95% confidence intervals for the golden lancehead pitviper, *Bothrops insularis*. Each interval represents an estimate between primary occasions *t* and *t*+1. The grey dashed line represents a stable population growth.

The population abundance for the sampling area varied between sampling periods from 80 to 218 individuals (Fig. 2). From 2002 to 2010 the population showed an average annual finite population growth rate of 0.93 (CI = 0.47–1.38), ranging from 0.4 to 2.1 (Fig. 3).

## Discussion

Although most previous estimates of snake survival did not account for detection probability [41]–[42], a general pattern of high survival was suggested for vipers [4], [43]–[44]. Here, survival for the golden lancehead ranged from 0.55 to 0.79, depending on season and sex. Such values are intermediate when compared to other viper population studies that accounted for imperfect detectability [9], [15], [44].

Our results indicate that season may have an effect on survival, although not pronounced. Different factors may explain the seasonal variation in survival. No evidence for a positive relationship between weather variation and the activity of the golden lancehead was found by Marques, Martins, Develey, Macarrão & Sazima [24] despite the fact that reptile activity usually correlates with weather variation [25]. Environmental variation, which includes harsh winters and droughts, may directly decrease individual survival, with impacts on population dynamics of snakes [45] and other vertebrates [46]–[47]. Survival may also be linked to prey availability, which is indirectly linked to a climatic seasonal regime. Food shortages may depend on weather stochasticity and dynamics of migratory bird movement in the Queimada Grande Island. As adults, the golden lancehead preys mostly upon two out of 41 migratory birds that are seasonally present on the island [24]. The tyrant flycatcher *Elaenia chilensis* is the most common prey found in the gut of adult pitvipers. This passerine bird appears on the coast of southeastern Brazil at the end of the wet season (austral summer), which coincides with the higher survival estimates of the snake [24]. Prey availability may play a special role on survival and population trends [45]. Additionally, prey availability may affect reproduction, leading *B. insularis* to experience lower breeding frequency than its mainland sister species, *B. jararaca* [23].

Since permanent emigration is confounded with mortality in most mark-recapture models [48], the illegal removal of individuals, suggested by Martins, Sawaya & Marques [22], could also be responsible for the observed variation in survival. This, in turn, would imply in an important removal rate on the island. If such illegal trade targets the largest, and thus the oldest individuals, fewer snakes will be in the population long enough to reach older age classes, thus increasing the proportion of younger snakes (e.g., [16], study on *Crotalus pricei*). Age specific mortality could be tested in the free ranging population or captive individuals using radiotelemetry and known fate models. Since body size is usually positively related with fecundity in female reptiles [49], the average population fecundity could also decrease, with impacts on population growth, such as those observed for the green python, *Morelia viridis* in Oceania [50]. Targeting adult females may be advantageous to establish colonies of captive bred animals or because of interest on meat or eggs (marine turtles, for instance [51]).

Sex-specific differences in survival were not strongly supported by our data but males presented slightly higher survival probabilities than females. This same pattern has been reported in the literature for other adult viper populations, including the asp viper, *Vipera aspis* [9] and non-viper snakes such as the eastern indigo snake [13]. *Bothrops insularis* is a potential prey of four raptors on the island [24], but predation seems not to be a strong pressure on the population [52]. Therefore, food and reproduction may represent the greatest obstacles to survive. Females, in this way, might pay a higher final cost to grow and breed than males because of the higher cost of producing vitellogenic follicles, possibly leading to higher survival probability for males.

The probability of temporary emigration was high in this study (∼0.70), and there was a high random probability of movement on and off the surveyed area among primary occasions (since cumulative QAIC*c* weight of Markovian type movement was low, 0.36), which suggests that sampling did not disturb animals. Although movement even for sedentary vertebrates such as pitvipers is expected at some point, the high temporary emigration might also result from the sampling design we used. Given the edge to interior ratio, transects may not be the best solution if movement is the aim of the study. Apart from the sampling design, investigators should be aware that not accounting for temporary emigration when it exists might result in imprecise estimation of vital parameters, including population abundance, number of recruits and survival [34]. Overall, the relevance of temporary emigration in snake studies remains poorly explored, since to our knowledge, no other studies so far have estimated the probability of temporary emigration [6].

Low detectability seems to be common in snake population studies [6], [9], [15], [53]–[54]. *Bothrops insularis* occurs in relatively high densities [22], [24] but detection was low. The brown tree snake, *Boiga irregularis* presented an estimated detection probability of 0.07 in a fenced 5-ha area [11]. Similarities in habitat (tropical systems) and habit (arboreal) of both snakes, *B. insularis* and *B. irregularis*, might lead to low detection since spotting individuals in three-dimensional habitats can be challenging due to the complex architecture of the forest. We did not observe any effect of temperature on detection probability. We hypothesize that the forest might lessen the heat gains and losses, protecting individuals from the windy conditions typical of the grassy areas, despite the more stable temperature of the subtropics. Detectability was also uncorrelated with body size, unlike in other snake species [15], [44]. Adults are relatively large (500–900 mm snout-vent length) and pale yellowish to brownish cream colored, which may reduce heterogeneity in detectability.

Trap-shyness has been reported for the rattlesnake *Crotalus horridus* that abandoned their shelters and became less prone to be recaptured [54]. Capturing also might disturb *C. pricei* [16] and partly reduced detectability of the aquatic viper *Agkistrodon piscivorus* [12]. Behavioral effects of capturing the golden lancehead were negligible (QAIC*c* cumulative weight was 0.34), although a negative effect was present showing that increasing manipulation of individuals may increase stress and a potential trap-shyness response. These results emphasize the importance of considering the best marking techniques and field procedures available, which can directly impact survival and behavior [55].

We estimated that from 80 to 218 adult individuals were available to be captured on each primary occasion in the sampling area. The finite population growth rate of *B. insularis* was also variable among years, with periods in which growth rate doubled to periods in which it decreased to half. On average, we estimated a cross-year growth rate of 0.93 (which translates to a decrease of 7%) during the study, but we cannot assure the population is declining because of the uncertainty around the estimates (the confidence intervals of our estimates included 1, which means stability).

The decline of the population suggested by [22] between 1995 and 2007, was attributed to illegal trade and, less importantly, to habitat reductions. *Bothrops insularis* is listed as critically endangered in the IUCN Red List because of its small distribution, the occurrence in a single location, and decreased habitat quality [21]. Generally, populations of more variable size face a higher extinction risk [56] and stochasticity may be crucial for the future of such populations [46], [57]. Thus, we should consider natural as well as man-induced oscillations as potential extinction drivers for such a small population. Actions to protect the species could involve law enforcement for a better protection of the island (e.g., to avoid illegal removal of snakes), and the protection of bird migratory routes for the species that use the island and mainland habitats, which are important to maintaining the availability of food resources for the snake.

The study of snake populations has gained more attention in the past years, but knowledge on population biology and dynamics of most species is still lacking. To our knowledge, the golden lancehead is the first Neotropical snake to have such information available while accounting for imperfect detection. This information is crucial and timely due to the sensitivity of an insular ecosystem and the possible illegal removal of snakes that threats this endemic species. Using robust statistical models we were unable to confirm the decline proposed by [22] although oscillations may be important for the dynamics of such species. Hence, we conclude that the population was stable during the study period, but the uncertainty observed here shows that caution is needed.

Basic knowledge gathered on natural history, together with estimates for population assessments and monitoring allows to further advance to a more comprehensible interpretation of such populations and their dynamics. In this sense, we recommend the use of robust tools such as mark-recapture, occupancy modeling or distance sampling to estimate vital rates. It may also be interesting to concentrate sampling effort in a smaller area and increase the number of secondary occasions in order to get more accurate vital rates estimates important to the conservation of target species.

## Supporting Information

Table S1 **Model selection results.** Model selection results. QAIC*c* = Akaike's information criteria with small sample size correction and adjusted for extra binomial variation, QΔAIC*c* = difference between top model and the current model, *w_i_* = QAIC*c* weights, *k* = number of parameters, Deviance = difference of the current model and the saturated model. Covariates for parameters are: Season – dry and wet; Sex – adult males and adult females; Mintemp – minimum temperature; (.) – constant parameter.
(DOCX)
