# Supplementary material for: Population Dynamics of the Critically Endangered Golden Lancehead Pitviper, Bothrops insularis: Stability or Decline?
Source: PLoS One. 2014 Apr 22;9(4):e95203. doi: 10.1371/journal.pone.0095203 (PMC3995933; doi:10.1371/journal.pone.0095203)
Supplement: Table S1 — Model selection results. Model selection results. QAICc = Akaike's information criteria with small sample size correction and adjusted for extra binomial variation, QΔAICc = difference between top model and the current model, wi = QAICc weights, k = number of parameters, Deviance = difference of the current model and the saturated model. Covariates for parameters are: Season – dry and wet; Sex – adult males and adult females; Mintemp – minimum temperature; (.) – constant parameter. (DOCX) [file pone.0095203.s001.docx]

**Supporting information**

Table 1

| Model | QAIC*c* | Δ QAICc | QAIC*c* Weights | *k* | QDeviance |
| --- | --- | --- | --- | --- | --- |
| Φ(season) γ"(.) p(.) | 630.71 | 0.00 | 0.08 | 4 | 622.59 |
| Φ(.) γ"(.) p(.) | 631.22 | 0.52 | 0.06 | 3 | 625.15 |
| Φ(.) γ"(.) p(.) γ’(.) | 631.93 | 1.22 | 0.05 | 4 | 623.81 |
| Φ(season) γ"(.) p(.) c(.) | 631.95 | 1.24 | 0.04 | 5 | 621.78 |
| Φ(season) γ"(.) p(.) γ’(.) | 632.21 | 1.50 | 0.04 | 5 | 622.03 |
| Φ(season+sex) γ"(.) p(.) | 632.33 | 1.63 | 0.04 | 5 | 622.16 |
| Φ(sex) γ"(.) p(.) | 632.40 | 1.70 | 0.04 | 4 | 624.29 |
| Φ(.) γ"(.) p(.) c(.) | 632.47 | 1.76 | 0.03 | 4 | 624.35 |
| Φ(season) γ"(.) p(mintemp) | 632.71 | 2.00 | 0.03 | 5 | 622.53 |
| Φ(season) γ"(.) p(svl) | 632.76 | 2.05 | 0.03 | 5 | 622.58 |
| Φ(.) γ"(.) p(mintemp) | 632.97 | 2.26 | 0.03 | 4 | 624.85 |
| Φ(sex) γ"(.) p(.) γ’(.) | 633.07 | 2.36 | 0.03 | 5 | 622.89 |
| Φ(.) γ"(.) p(svl) | 633.26 | 2.55 | 0.02 | 4 | 625.14 |
| Φ(.) γ"(.) p(.) γ’(.) c(.) | 633.33 | 2.62 | 0.02 | 5 | 623.16 |
| Φ(season) γ"(.) p(.) γ’(.) c(.) | 633.52 | 2.81 | 0.02 | 6 | 621.27 |
| Φ(season+sex) γ"(.) p(.) c(.) | 633.60 | 2.89 | 0.02 | 6 | 621.35 |
| Φ(sex) γ"(.) p(.) c(.) | 633.66 | 2.95 | 0.02 | 5 | 623.48 |
| Φ(.) γ"(.) p(mintemp) γ’(.) | 633.82 | 3.11 | 0.02 | 5 | 623.64 |
| Φ(season+sex) Γ”(.) p(.) γ’(.) | 633.89 | 3.18 | 0.02 | 6 | 621.64 |
| Φ(.) γ"(.) p(svl) γ’(.) | 633.98 | 3.27 | 0.02 | 5 | 623.80 |
| Φ(season) γ"(.) p(mintemp) c(.) | 633.99 | 3.28 | 0.02 | 6 | 621.74 |
| Φ(season) γ"(.) p(svl) c(.) | 634.02 | 3.31 | 0.02 | 6 | 621.77 |
| Φ(sex) γ"(.) p(mintemp) | 634.16 | 3.45 | 0.01 | 5 | 623.98 |
| Φ(season) γ"(.) p(mintemp) γ’(.) | 634.24 | 3.54 | 0.01 | 6 | 622.00 |
| Φ(sex) γ"(.) p(svl) | 634.27 | 3.56 | 0.01 | 5 | 624.09 |
| Φ(season) γ"(.) p(svl) γ’(.) | 634.27 | 3.56 | 0.01 | 6 | 622.02 |
| Φ(season+sex) γ"(.) p(svl) | 634.28 | 3.57 | 0.01 | 6 | 622.03 |
| Φ(season+sex) γ"(.) p(mintemp) | 634.32 | 3.62 | 0.01 | 6 | 622.08 |
| Φ(.) γ"(.) p(mintemp) c(.) | 634.51 | 3.80 | 0.01 | 5 | 624.33 |
| Φ(sex) γ"(.) p(.) γ’(.) c(.) | 634.51 | 3.80 | 0.01 | 6 | 622.26 |
| Φ(.) γ"(.) p(svl) c(.) | 634.52 | 3.81 | 0.01 | 5 | 624.34 |
| Φ(season) γ"(.) p(mintemp+svl) | 634.77 | 4.06 | 0.01 | 6 | 622.52 |
| Φ(sex) γ"(.) p(svl) γ’(.) | 634.95 | 4.25 | 0.01 | 6 | 622.71 |
| Φ(sex) γ"(.) p(mintemp) γ’(.) | 634.97 | 4.26 | 0.01 | 6 | 622.72 |
| Φ(.) γ"(.) p(svl) p (mintemp) | 635.02 | 4.31 | 0.01 | 5 | 624.84 |
| Φ(season+sex) γ"(.) p(.) γ’(.) c(.) | 635.22 | 4.51 | 0.01 | 7 | 620.88 |
| Φ(.) γ"(.) p(svl) γ’(.) c(.) | 635.40 | 4.69 | 0.01 | 6 | 623.15 |
| Φ(.) γ"(.) p(mintemp) γ’(.) c(.) | 635.40 | 4.70 | 0.01 | 6 | 623.16 |
| Φ(season) γ"(.) p(mintemp) γ’(.) c(.) | 635.56 | 4.85 | 0.01 | 7 | 621.22 |
| Φ(season+sex) γ"(.) p(svl) c(.) | 635.60 | 4.89 | 0.01 | 7 | 621.27 |
| Φ(season) γ"(.) p(svl) γ’(.) c(.) | 635.60 | 4.89 | 0.01 | 7 | 621.27 |
| Φ(sex) γ"(.) p(svl) c(.) | 635.60 | 4.89 | 0.01 | 6 | 623.35 |
| Φ(season+sex) γ"(.) p(mintemp) c(.) | 635.66 | 4.95 | 0.01 | 7 | 621.33 |
| Φ(sex) γ"(.) p(mintemp) c(.) | 635.72 | 5.01 | 0.01 | 6 | 623.47 |
| Φ(season+sex) γ"(.) p(svl) γ’(.) | 635.86 | 5.15 | 0.01 | 7 | 621.52 |
| Φ(.) γ"(.) p(mintemp+svl) γ’(.) | 635.88 | 5.18 | 0.01 | 6 | 623.64 |
| Φ(season+sex) γ"(.) p(mintemp) γ’(.) | 635.91 | 5.21 | 0.01 | 7 | 621.58 |
| Φ(season) γ"(.) p(mintemp+svl) c(.) | 636.07 | 5.36 | 0.01 | 7 | 621.74 |
| Φ(sex) γ"(.) p(mintemp+svl) | 636.08 | 5.37 | 0.01 | 6 | 623.83 |
| Φ(season+sex) γ"(.) p(mintemp+svl) | 636.29 | 5.59 | 0.01 | 7 | 621.96 |
| Φ(season) γ"(.) p(mintemp+svl) γ’(.) | 636.32 | 5.61 | 0.01 | 7 | 621.99 |
| Φ(sex) γ"(.) p(svl) γ’(.) c(.) | 636.46 | 5.75 | 0.00 | 7 | 622.13 |
| Φ(.) γ"(.) p(svl) c(.) p (mintemp) | 636.58 | 5.87 | 0.00 | 6 | 624.33 |
| Φ(sex) γ"(.) p(mintemp) γ’(.) c(.) | 636.60 | 5.89 | 0.00 | 7 | 622.26 |
| Φ(sex) γ"(.) p(mintemp+svl) γ’(.) | 636.89 | 6.19 | 0.00 | 7 | 622.56 |
| Φ(season+sex) γ"(.) p(svl) γ’(.) c(.) | 637.23 | 6.52 | 0.00 | 8 | 620.80 |
| Φ(season+sex) γ"(.) p(mintemp) γ’(.) c(.) | 637.28 | 6.57 | 0.00 | 8 | 620.85 |
| Φ(.) γ"(.) p(mintemp+svl) γ’(.) c(.) | 637.48 | 6.77 | 0.00 | 7 | 623.15 |
| Φ(season) γ"(.) p(mintemp+svl) γ’(.) c(.) | 637.64 | 6.94 | 0.00 | 8 | 621.22 |
| Φ(season+sex) γ"(.) p(mintemp+svl) c(.) | 637.67 | 6.96 | 0.00 | 8 | 621.24 |
| Φ(sex) γ"(.) p(.) c(.) p (mintemp+svl) | 637.67 | 6.96 | 0.00 | 7 | 623.34 |
| Φ(season+sex) γ"(.) p(mintemp+svl) γ’(.) | 637.90 | 7.19 | 0.00 | 8 | 621.47 |
| Φ(sex) γ"(.) p(mintemp+svl) γ’(.) c(.) | 638.56 | 7.85 | 0.00 | 8 | 622.13 |
| S(season+sex) γ"(.) γ’(.) p(mintemp+svl) c(mintemp+ svl) | 639.31 | 8.60 | 0.00 | 9 | 620.77 |
